# Simultaneous determination of eight major bioactive compounds in Dachengqi Tang (DT) by high-performance liquid chromatography

**DOI:** 10.1186/1749-8546-3-5

**Published:** 2008-04-29

**Authors:** Wenfu Tang, Meihua Wan, Zhengyan Zhu, Guanyuan Chen, Xi Huang

**Affiliations:** 1 Department of Integrative Traditional Chinese and Western Medicine, West China Hospital, Sichuan University, Chengdu 610041, Sichuan, China

## Abstract

**Background:**

*Dachengqi Tang *(DT) is a common traditional Chinese medicine formula for expelling *neire *('internal heat') in the stomach and intestines. There was no reliable analytical method available for the quality control of DT.

**Methods:**

A high-performance liquid chromatography (HPLC) method with a reverse phase C_18 _column (150 × 4.6 mm) was developed. The mobile phase was methanol with 0.2% acetic acid. Eight markers including naringin, hesperidin, aloe emodin, rhein, honokiol, magnolol, emodin and chrysophanol were determined.

**Results:**

Regression analysis revealed a linear relationship between the concentrations of the markers and the peak area ratio of the standards and internal standard. The limit of detection (S/N = 3) and the limit of qualification (RSD < 20%) ranged from 0.21 to 0.43 ng/μl and 0.76 to 1.74 ng/μl respectively. The recovery was between 95.6% and 103.4%. The tests on the samples from three batches of DT showed that the profiles of the markers did not vary significantly among batches.

**Conclusion:**

A reliable HPLC method for simultaneous determination of the eight markers in DT was developed.

## Background

Composed of *Radix et Rhizoma Rhe*i (*Dahuang*), *Cortex Magnoliae Officinalis (Houpo)*, *Fructus Aurantii Immaturus (Zhishi) *and *Natrii Sulfas *(*Mangxiao*) at a ratio of 1:1:1:1, *Dachengqi Tang *(DT) is a purgative Chinese herbal decoction for expelling *neire *('internal heat') in the stomach and intestines [1]. This formula is often used to treat acute pancreatitis, dysentery, cholecystitis, cholelithiasis, cholangitis, ileus, peritonitis and abdominal distention [2]. Acute pancreatitis is one of the most serious acute abdominal disorders for which conventional medicines are not effective. DT has been used in China to treat acute pancreatitis for over 25 years [3]. DT was demonstrated to promote gastrointestinal motility and inhibit cytokine activities and immune/inflammatory response in acute pancreatitis and ileus [4,5].

While the active components in DT remain to be confirmed, several bioactive compounds isolated from an individual component have been identified. Chen *et al*. reported that emodin from rhubarb modulated the Ca^2+ ^signal transduction of smooth muscle cells in multiple-organ dysfunction syndrome [6]. Emodin was shown to be an active antibacterial agent [7] and beneficial to acute pancreatitis [8]. Aloe emodin and chrysophanol were detected in rats after treatment with DT [9]. Rhein and emodin, which are the active metabolites of rhubarb, were found to inhibit lipid peroxide production and scavenge and/or inhibit hydroxyl radicals [10,11]. Naringin and hesperidin extracted from immature bitter oranges demonstrated antimicrobial [12] and hypoglycemic effects [13]. Kawaguchi *et al*. reported that hesperidin from immature bitter orange inhibited the lipases from porcine pancreas and *Pseudomonas *[14]. Furthermore, honokiol and magnolol from magnolia bark exerted prokinetic and inhibitory effects on gastrointestinal movement [15] and on streptococcal glucosyltransferases [16] respectively.

**Figure 1:**
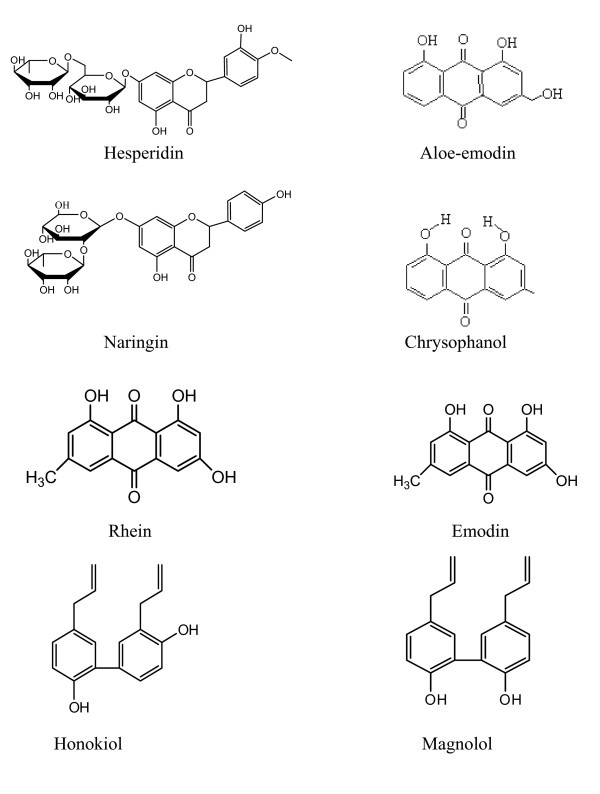
The chemical structures of eight markers in the present study.

Previously, six markers from DT were simultaneously analyzed with iso-gradient method [17]. In the present study, we developed a high-performance liquid chromatography (HPLC) method for simultaneous determination of eight markers from DT including aloe emodin, rhein, emodin and chrysophanol from *Radix et Rhizoma Rhe*i, honokiol and magnolol from *Cortex Magnoliae Officinalis*, naringin and hesperidin from *Fructus Aurantii Immaturus *(Figure 1).

## Methods

### Material and reagents

The reference standards of naringin, hesperidin, aloe emodin, rhein, honokiol, magnolol, emodin, chrysophanol and 1, 8-dihydroxyanthraquinone, were purchased from the National Institute for the Control of Pharmaceutical and Biological Products of China. The purity of all reference standards was above 98%. Methanol and acetic acid were purchased from TEDIA (USA) and Chongqing Chemistry Co Ltd (China) respectively. Granules of *Radix et Rhizoma Rhe*i, *Cortex Magnoliae Officinalis*, *Fructus Aurantii Immaturus *and *Natrii Sulfas *were purchased from Chengdu Green Pharmacy Co Ltd (China). All reagents used in the study were of HPLC grade. Ultra-pure water used for the mobile phase was prepared by an ultra-pure water purification system (Shanghai Anting Co Ltd, China).

### Sample preparation

Each *DT *granule sample (5 g) was ground to fine powder (80 meshes). One hundred milligrams of the fine powder was accurately weighed and dissolved in 50 ml of 65% (v/v) aqueous methanol solution. The internal standard (1, 8-dihydroxyanthraquinone, 1 ml, 5 mg/ml) was added to the solution which was then extracted in an ultrasonic water bath (70°C) for 120 min. The sample solution was centrifuged (× 3000 g) for 15 min and the supernatant was filtered through a 0.45 μm Millipore membrane filter (Millipore, USA).

### Instrument and chromatographic conditions

All separations were performed on a Waters HPLC system equipped with a binary pump, auto-sampler and Waters 2487 dual λ absorbance detector (Waters, USA). A RP-C_18 _HPLC column (150 × 4.6 mm, S-5 μm, 12 nm) and a guard column (GL Sciences, Japan) were used. Data were processed on a Waters Empower chromatographic workstation (Waters, USA).

The mobile phase was a mixture of solvent (A) 100% methanol and solvent (B) 0.2% aqueous acetic acid (pH 3.12, 1:500, v/v) (A-B: 20 min 36:64; 19 min 65:35; 21 min 70:30). The flow rate was 1.0 ml/min and injection volume was 10 μl. A ten min interval was given between sample injections. The column effluents were simultaneously monitored at 254 nm (for aloe emodin, rhein, chrysophanol and emodin) and 280 nm (for honokiol, magnolol, naringin and hesperidin). The column temperature was controlled at 26°C.

### Optimization of chromatographic conditions

While it is difficult to separate naringin and hesperidin, honokiol and magnolol, aloe emodin, rhein, emodin and chrysophanol as they are structurally similar, separation was improved when 0.2% (1:500, v/v) aqueous acetic acid was added to the sample solution. A mixture of water and methanol was chosen for the separation as all eight markers dissolve in both water and methanol. The ratio 38:62 (v/v) of methanol and water was optimal for the separation of hesperidin and naringin, and 65:35 (v/v) for the separation of the other six markers, hence the above mentioned gradient elution program. The HPLC column of C_18 _(150 × 4.6 mm) was chosen to ensure the run time to be within 60 min.

### Calibration

All calibration curves were plotted based on linear regression analysis of the concentrations of the eight markers (x) versus the peak area ratio (y). The concentration of the internal standard was 100 μg/ml for all markers. Each calibration curve was obtained with six concentrations in triplicates.

The standard solutions of the eight markers were diluted with 65% aqueous methanol to provide appropriate concentrations with the internal standard (100 μg/ml). The diluted solutions were injected three times. The quantity of each marker was determined according to the corresponding calibration curve. The limit of detection (LOD) for each marker was determined when the peak signal-to-noise ratio was at 3. The limit of quantification (LOQ) for each marker was determined when the RSD of the peak's quantity from the corresponding calibration curve was less than 20%.

### Precision

Intra-day and inter-day variability was studied to evaluate the precision of the method. Three solutions (high, medium and low concentrations) of the eight markers with the internal standard (100 μg/ml) were prepared. The quantity of each component was calculated from the corresponding calibration curve. The relative standard deviation (RSD) was taken as the measure of precision. The inter-day reproducibility test was carried out on three different days.

### Recovery

Three known quantities of the eight markers were added to 50 ml of 65% aqueous methanol solution with 1 ml of the internal standard (5 mg/ml) and the powder of DT (400 mg). The resultant samples were extracted and analyzed as described above. The quantification of each marker was obtained according to the corresponding calibration curve.

### Statistical analysis

Statistical analysis was performed with the PEMS3.1 for Windows (Sichuan University, China). The quantitative results were presented as mean with standard deviation if the distribution was normal and as maximum, median and minimum if the data distribution was not normal. Linear regression for the calibration curves was tested. Single factor ANOVA was performed on each component for their determination in the three batches of DT. The comparison of two independent samples was evaluated with the Student's t-test when the distribution was normal. Test result was considered to be statistically significant when its two-tailed P value was less than 0.05.

## Results

### Chromatography

**Figure 2:**
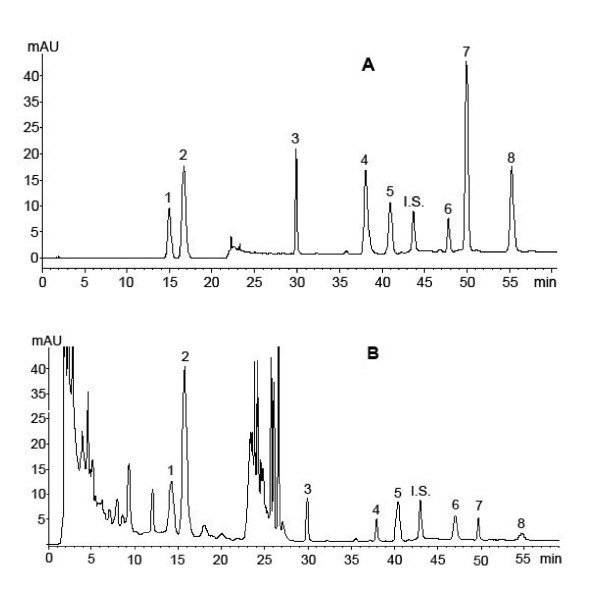
**A **A chromatogram of pure standards: (1) naringin, (2) hesperidin (3) aloe emodin, (4) rhein, (5) honokiol, (6) magnolol, (7) emodin, (8) chrysophanol and the internal standard (IS), 1, 8-dihydroxyanthraquinone. **B **Simultaneous determination of eight markers: (1) naringin, (2) hesperidin (3) aloe emodin, (4) rhein, (5) honokiol, (6) magnolol, (7) emodin, (8) chrysophanol and the internal standard (IS), 1, 8-dihydroxyanthraquinone, in a Dachengqi Tang sample.

A chromatogram of standard naringin, hesperidin, aloe emodin, rhein, honokial, magnolol, emodin and chrysophanol is shown in Figure 2A. These standards are well resolved with relatively high sensitivities at 14.484, 16.429, 30.065, 37.355, 40.579, 47.373, 49.060 and 54.463 min. The internal standard is separated at 43.309 min. The peaks are well separated from each other (peak purity > 95%) and show a characteristic profile.

### Linearity, precision and accuracy

All eight calibration curves displayed good linear relationships under the present chromatographic conditions (Table 1). The LOD and LOQ were in the ranges of 0.21–0.43 ng/μl and 0.76–1.74 ng/μl respectively for all eight markers. The recovery tests of all eight markers were performed by adding the standards to the powder. The results indicated that the recovery for all eight markers were in the range of 95–104% (Table 2). The overall intra- and inter-day variability was less than 4.0% for all eight markers (Tables 3 and 4).

**Table 1:** Calibration of eight markers in *Dachengqi Tang*

Marker	Calibration	r^2^	P	Test range (μg/ml)	LOD (μg/ml)	LOQ (μg/ml)
Naringin	*y *= 0.2009 *x *- 0.2487	0.9996	<0.001	2.50–200	0.31	0.93
Hesperidin	*y *= 0.1996 *x *- 1.3249	0.9982	<0.001	6.95–556	0.43	1.74
Aloe emodin	*y *= 0.1794 *x *- 0.267	0.9994	<0.001	1.83–146	0.23	0.92
Rhein	*y *= 0.1728 *x *- 0.039	0.9996	<0.001	1.75–140	0.21	0.84
Honokial	*y *= 0.1388 *x *- 0.0792	0.9992	<0.001	1.05–84	0.25	0.81
Magnolol	*y *= 0.1363 *x *+ 0.0025	1.0000	<0.001	2.07–166	0.23	1.03
Emodin	*y *= 0.4250 *x *+ 0.0452	0.9992	<0.001	2.20–176	0.28	0.83
Chrysophanol	*y *= 0.2125 *x *– 0.0184	0.9998	<0.001	2.05–164	0.25	0.76

*y*: peak area ratio (analyte/internal standard); x: concentration of analyte (μg/ml); LOD (limit of detection): S/N = 3; LOQ (limit of quantification): RSD < 20%

**Table 2:** Recovery of eight markers in *Dachengqi Tang *samples (n = 3)

Marker	Concentrations (μg/ml)	Concentration measured (μg/ml, mean (SD))	Recovery^a ^(%)	RSD ^b ^(%)
Naringin	5.0	4.87 (0.09)	97.4	1.8
	50.0	50.28 (1.26)	100.6	2.5
	150.0	148.95 (1.81)	99.3	1.2
Hesperidin	13.9	13. 79 (0.38)	99.2	2.8
	139.0	142.42 (2.26)	102.5	1.6
	417.0	419.84 (3.32)	100.7	0.7
Aloe emodin	3.66	3.76 (0.11)	102.7	2.9
	36.6	36.17 (1.15)	98.8	3.2
	109.8	111.73 (3.29)	101.8	2.9
Rhein	3.5	3.41 (0.09)	97.4	2.6
	35.0	33.47 (0.96)	95.6	2.9
	105.0	107.68 (1.41)	102.6	1.3
Honokial	2.1	2.15 (0.07)	97.6	3.3
	10.5	10.18 (0.23)	97.0	2.3
	60.3	61.24 (1.32)	101.6	2.2
Magnolo	l 4.2	4.16 (0.11)	99.0	2.6
	42.0	43.42 (1.24)	103.4	2.9
	126.0	126.61 (1.86)	100.5	1.5
Emodin	2.2	2.24 (0.05)	101.8	2.2
	22.0	21.54 (0.53)	97.9	2.5
	66.0	67.28 (1.23)	101.9	1.8
Chrysophanol	2.06	2.11 (0.06)	102.4	2.8
	20.6	20.15 (0.21)	97.8	1.0
	61.8	63.42 (1.26)	102.6	2.0

^a ^Recovery (%) = mean of measured concentration/added concentration × 100

^b ^RSD (%) =(SD/mean) × 100

**Table 3:** Intra-day variability for the simultaneous determination of eight markers in *Dachengqi Tang *(n = 6)

Marker	Concentration (μg/ml)	Measured concentration (μg/ml, mean (SD))	Accuracy^a ^(%)	RSD^b ^(%)
Naringin	25.0	24.87 (0.29)	99.5	1.2
	62.5	62.80 (1.26)	100.5	2.0
	156.3	155.53 (3.02)	99.5	1.9
Hesperidin	69.5	69. 79 (1.38)	100.4	2.0
	173.8	172.14 (3.62)	99.0	2.1
	434.4	429.38 (4.62)	98.8	1.1
Aloe emodin	18.3	18.72 (0.53)	102.3	2.8
	45.8	46.79 (1.58)	102.2	3.4
	114.4	112.77 (3.56)	98.6	3.2
Rhein	17.5	17.81 (0.21)	101.8	1.2
	43.8	43.83 (0.69)	100.1	1.6
	109.4	108.86 (1.55)	99.5	1.4
Honokial	10.5	10.25 (0.11)	97.6	1.0
	26.3	26.18 (0.22)	99.5	0.8
	65.6	66.24 (1.53)	101.0	2.3
Magnolol	21.0	21.06 (0.47)	100.3	2.2
	52.5	53.10 (1. 58)	101.1	3.0
	131.3	131.16 (2.06)	99.9	1.6
Emodin	11.0	11.39 (0.24)	103.5	2.1
	27.5	26.54 (0.71)	96.5	2.7
	68.8	68.50 (1.34)	99.6	1.9
Chrysophanol	10.3	10.52 (0.23)	102.1	2.2
	25.8	25.57 (0.41)	99.1	1.6
	64.4	65.55 (1.17)	101.8	1.8

^a ^Accuracy (%) = mean of measured concentration/added concentration × 100

^b ^RSD (%) (relative standard deviation) = (SD/mean) × 100

**Table 4:** Inter-day variability for the simultaneous determination of eight markers in *Dachengqi Tang *(n = 3)

Compounds	Concentration (μg/ml)	Measured concentration (μg/ml, mean (SD))	Accuracy^a ^(%)	RSD^b ^(%)
Naringin	25.0	24.83 (0.36)	99.3	1.4
	62.5	62.68 (0.97)	100.3	1.5
	156.3	157.72 (4.21)	100.9	2.7
Hesperidin	69.5	69. 07 (2.68)	99.4	3.9
	173.8	170.49 (4.35)	98.1	2.5
	434.4	428.44 (2.96)	98.7	0.7
Aloe emodin	18.3	18.47 (0.32)	100.9	1.7
	45.8	44.73 (0.86)	97.7	1.9
	114.4	113.89 (2.31)	99.6	2.0
Rhein	17.5	17.78 (0.19)	101.6	1.1
	43.8	44.32 (0.94)	101.2	2.1
	109.4	109.06 (0.87)	99.7	0.8
Honokial	10.5	10.67 (0.17)	101.6	1.6
	26.3	25.89 (0.92)	98.4	3.5
	65.6	67.86 (2.23)	103.4	3.3
Magnolol	21.0	21.63 (0.71)	103.0	3.2
	52.5	52.60 (0.85)	101.9	1.6
	131.3	130.91 (2.64)	99.7	2.0
Emodin	11.0	11.46 (0.40)	104.2	3.5
	27.5	27.23 (0.87)	99.0	3.2
	68.8	68.32 (1.46)	99.3	2.1
Chrysophanol	10.3	10.65 (0.31)	103.4	2.9
	25.8	24.98 (0.44)	96.8	1.8
	64.4	66.24 (1.32)	102.9	2.0

^a ^Accuracy (%) = mean of measured concentration/nominal concentration × 100

^b ^RSD (%) = (SD/mean) × 100

### Determination of the eight markers in DT

The method described above was subsequently applied to the simultaneous qualification of the eight markers in three batches of commercial DT samples. The data of the three batches of samples are highly consistent (Figure 2B and Table 5). The present method is feasible and reliable for simultaneous determination of many anthraquinone, flavone and neolignan compounds in complex Chinese medicine formulae such as DT.

**Table 5:** Simultaneous determination of eight markers in three commercial samples of *Dachengqi Tang*

Marker	Content (mg/g)		
	Batch 1	Batch 2	Batch 3
Naringin	3.89 (0.07)	3.82 (0.07)	3.79 (0.06)
Hesperidin	11.27 (0.23)	11.04 (0.22)	10.87 (0.22)
Aloe emodin	1.72 (0.08)	1.69 (0.07)	1.77 (0.08)
Rhein	0.88 (0.05)	0.83 (0.03)	0.86 (0.05)
Honokial	1.25 (0.08)	1.28 (0.07)	1.24 (0.08)
Magnolol	1.06 (0.07)	1.10 (0.08)	1.16 (0.06)
Emodin	2.39 (0.16)	2.54 (0.11)	2.50 (0.11)
Chrysophanol	0.52 (0.03)	0.57 (0.01)	0.55 (0.01)

Values are mean (SD) n = 3. Single factor ANOVA was performed on each component. No significant difference was observed in the measured concentration of each marker in three batches of DT.

## Discussion

We developed an analytical method for simultaneous determination of eight markers in DT by HPLC, which is more useful than the previous method with six markers [17]. The two additional markers, namely naringin and hesperidin, are the main therapeutic ingredients of the formula.

Apart from these eight markers, there are many other ingredients to be identified and tested with various analytical methods including mass spectrometry. Moreover, we studied only the local herbs from Sichuan, China; it would be necessary to compare herbs from various regions in China with the present HPLC method.

## Conclusion

We developed a reliable HPLC method for simultaneous determination of eight markers in DT, namely naringin, hesperidin, aloe emodin, rhein, honokial, magnolol, emodin and chrysophanol.

## Competing interests

The authors declare that they have no competing interests.

## Authors' contributions

XH conceived the idea of the study. MW and ZZ performed sample collection, method development, validation, and sample analysis. WT and GC participated in the design of the study, collection of chemical markers, and drafting of the manuscript. All authors read and approved the final manuscript.
